# It Started With a Kiss: Monitoring Organelle Interactions and Identifying Membrane Contact Site Components in Plants

**DOI:** 10.3389/fpls.2020.00517

**Published:** 2020-05-06

**Authors:** Alice L. Baillie, Anna-Lena Falz, Stefanie J. Müller-Schüssele, Imogen Sparkes

**Affiliations:** ^1^School of Biological Sciences, University of Bristol, Bristol, United Kingdom; ^2^Institut für Nutzpflanzenforschung und Ressourcenschutz (INRES), Rheinische Friedrich-Wilhelms-Universität Bonn, Bonn, Germany

**Keywords:** membrane contact sites, organelle interactions, Förster resonance energy transfer, optical tweezers, tethers

## Abstract

Organelle movement and interaction are dynamic processes. Interpreting the functional role and mechanistic detail of interactions at membrane contact sites requires careful quantification of parameters such as duration, frequency, proximity, and surface area of contact, and identification of molecular components. We provide an overview of current methods used to quantify organelle interactions in plants and other organisms and propose novel applications of existing technologies to tackle this emerging topic in plant cell biology.

## Introduction

Membrane contact sites (MCS) are regions at which transient, physical interactions between organelles occur. These interactions allow exchange of molecules such as signals (e.g., calcium, Csordás et al., 2010) and membrane lipids (Michaud and Jouhet, 2019), and are important for regulating the number and positioning of some organelle types (Phillips and Voeltz, 2016; Mueller-Schuessele and Michaud, 2018; White et al., 2020). Direct molecular exchanges between organelles may alternatively be carried out by vesicle-mediated delivery (Scorrano et al., 2019) or by transient interaction between organelles of the same type (e.g., “kiss and run” in mitochondria, Liu et al., 2009). These processes involve the fusion of membranes and are therefore distinct from transient tethering at MCS, which by definition does not involve membrane fusion.

In recent years rapid progress has been made in understanding yeast and mammalian MCS, but studies in plants are less advanced. Some MCS roles will be unique to plants due to the existence of plant-specific compartments, metabolic pathways and processes such as plastids, photorespiration, light/dark adaption and stress responses (Oikawa et al., 2015; Pérez-Sancho et al., 2016).

Here we consider how to define, detect and quantify organelle interactions. We review established techniques for characterizing plant MCS and their protein components, as well as methods that have only been used in non-plant systems to date. For a review of the role of lipids in interactions at MCS, see Petit et al. (2019).

## Defining and Detecting Membrane Contact Sites

Identification of MCS requires that we define them in a measurable way. Multiple, functionally distinct types of contact site can form between given organelle pairs (Siao et al., 2016), with unique lipid/protein composition, and characteristic spatial and dynamic properties. Quantifying differences in contact site morphology and dynamics under different environmental conditions can provide clues to MCS function. Direct evidence for a functional, physical interaction requires demonstration of altered molecular exchange between the organelle pair in response to perturbation of MCS formation.

### Characterizing Contacts

**FIGURE 1:**
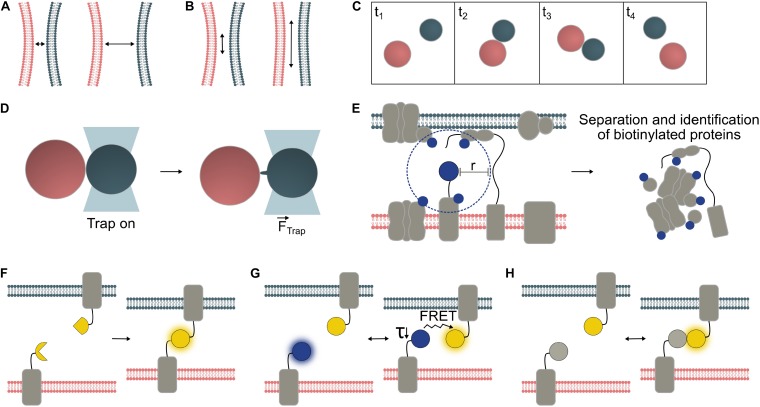
Temporal and spatial characterization of organelle interactions. Organelles are typically within 10 – 30 nm of one another at contact sites, though greater tethering distances have also been reported **(A)**. The surface area of the contact site varies depending on MCS type and in response to environmental conditions **(B)**. Frequency and duration of organelle interactions can be measured from microscopy time series. In this example, *Frequency* = 1 interaction/frame area/4 × frame length and *Duration* = 2 × frame length **(C)**. Biophysical techniques can be employed to probe physical interactions between organelles. Optical tweezers have confirmed physical interaction between organelle pairs including interaction between chloroplasts (red) and peroxisomes (gray), which may be mediated by peroxules, peroxisomal membrane extensions (Gao et al., 2016) **(D)**. Proteins with specific roles are enriched at MCS. Where one MCS-specific protein is known, proximity-labeling can be used to identify further MCS proteins. A biotin ligase (e.g., TurboID, Arora et al., 2019) fused to the protein of interest, biotinylates proteins within a given radius (“*r”*); for BioID *in vivo*, *r* was determined to be ∼10 nm (Kim et al., 2014) **(E)**. Various fluorescent sensor approaches may be used to visualize MCS (F-H). Bimolecular fluorescence complementation (BiFC) reporter systems emit a fluorescent signal upon the irreversible binding of split protein fragments to form the mature fluorescent protein **(F)**. FRET pairs (Förster resonance energy transfer) interact reversibly. Either the ratio between acceptor and donor fluorescence, or the decreased lifetime (τ) of the donor molecule can be measured to detect interactions **(G)**. Dimerization-dependent fluorescent proteins (ddFPs) emit a fluorescent signal only upon their interaction but, unlike BiFC reporters, interact reversibly **(H)**.

It is widely reported that interacting organelles reside within 10–30 nm of one another (Figure 1A), although tethering over distances up to 300 nm has been reported between mitochondria and the plasma membrane in yeast cells (Klecker et al., 2013; Scorrano et al., 2019). Organelle proximity at MCS will depend on the size and arrangement of tethering proteins. Organelles may also be brought close together through random collisions that are not indicative of interaction (Feng and Kornmann, 2018), especially in vacuolated plant cells in which the cytoplasmic void volume is small relative to the size of the cell. Since the proximity of juxtaposed organelles at MCS is generally much smaller than the maximum resolution of conventional light microscopes (∼200–250 nm), it is impossible to accurately measure the distance between organelles using this technique or to determine whether MCS formation could have occurred. Homotypic interactions present the additional challenge of distinguishing transient membrane contacts from fission or fusion events.

Contact surface area varies depending on the MCS in question (Figure 1B) and can change during development (McFarlane et al., 2017), or in response to biotic (Caplan et al., 2015) or abiotic stresses (Jaipargas et al., 2016; Lee et al., 2019). Changes in total MCS area can be mediated by changes in MCS abundance and/or size. For instance, abundance of a specific type of Endoplasmic reticulum-plasma membrane (ER-PM) contact site in Arabidopsis changed during cell maturation, whereas the average size of parallel membrane stretches (distance <15 nm) quantified via TEM remained largely unchanged at c. 160 nm (McFarlane et al., 2017). In quantitative TEM analyses, inter-organellar distance thresholds are often used to define MCS and quantify contact area between membranes (Naon et al., 2016; McFarlane et al., 2017). While this may yield a reasonable approximation for MCS number/size, proximity of two membranes is not direct evidence for a functional MCS.

The duration and frequency of MCS formation events (Figure 1C) have been less well studied than their spatial characteristics, probably due to the greater technical challenges of quantifying these parameters. Brief and/or infrequent interactions may be hard to capture, while long-lasting interactions may be difficult to monitor for their duration without sample drift and/or fluorescence bleaching problems. Hypothetically, random organelle collisions might be distinguished from regulated membrane interactions by measuring interaction duration, though brief juxtaposition does not necessarily preclude interaction. The dynamics of a given organelle interaction are likely to depend on the functional role of the contact. Varying physiological conditions will alter the demand for exchange of different molecule types, potentially affecting membrane contact frequency and or duration (Helle et al., 2013).

### Functional Criteria: Tethering and Molecular Exchange

While measurements of the parameters above (proximity, surface area, and duration/frequency) cannot provide proof that a functional MCS has formed, each may provide a reasonable basis for further investigation. Evidence of physical tethering and/or molecular exchange is necessary to confirm an interaction. When organelles interact, physical tethers form between them, increasing the force required for their separation (Figure 1D). This is challenging to measure but, techniques such as optical tweezers and shock waves generated from a focused femtosecond laser (see below) can allow demonstration of an increased separation force, which provides biophysical evidence for physical membrane contact (Sparkes, 2016, 2018; Oikawa et al., 2015). Direct demonstration of molecular exchange is another way to confirm a functional organelle interaction (Jouhet et al., 2004; Mehrshahi et al., 2013). Identification of proteins specific to the contact site, such as tethering proteins, functional proteins (e.g., channel proteins) and associated regulatory proteins, can shed light on MCS function and aid visualization through tagging of these MCS components with fluorescent proteins (FPs) (McFarlane et al., 2017). However, many MCS involve multiple tethering proteins, which can prevent mutation of an individual tether from having a measurable phenotypic effect (Scorrano et al., 2019).

## Techniques Used to Study Organelle Interactions

### Imaging Organelle Dynamics and MCS

While close organelle proximity is insufficient grounds to confirm an interaction, characterizing juxtaposition duration/frequency (Oikawa et al., 2015; Gao et al., 2016), correlation of movement (Sinclair et al., 2009; Barton et al., 2013; Higa et al., 2014) and contact area (Lee et al., 2019) remains useful, especially when investigating the effects of different environmental treatments (Jaipargas et al., 2016) or manipulating putative tether expression (Mueller and Reski, 2015). Confocal microscopy has become the primary tool of choice, with the wide palate of available fluorophores allowing simultaneous visualization of multiple organelles and proteins (Valm et al., 2017). Electron microscopy (EM) can offer higher resolution, and FPs can be detected using immunogold (Pérez-Sancho et al., 2015; Cui et al., 2016; Wang et al., 2016) or Correlative light and electron microscopy (CLEM), although these approaches are low-throughput. While EM requires sample fixation and therefore abolishes system dynamics, it allows much more accurate quantification of organelle proximity (Caplan et al., 2015) and MCS area, especially if tomography approaches are used to allow 3D reconstruction (Wang et al., 2019).

Where MCS tether proteins are known (see below), FP fusions can be created to specifically label MCS, or antibodies raised against tethers for immunogold EM. Lee et al. (2019) tagged known Arabidopsis ER-PM tether Syt1 (Pérez-Sancho et al., 2015) with GFP and compared the localization of this signal with that of MAPPER-GFP, a more ubiquitous ER-PM contact marker adapted from mammalian systems. This demonstrated that the subset of ER-PM contact sites containing SYT1 were involved in increasing ER-PM contact in response to ionic stress.

Super-resolution microscopy techniques use various innovative approaches to overcome the diffraction barrier, allowing imaging of biological structures in greater detail than by conventional light microscopy. Use of these techniques to study MCS components in plants has so far been very limited, though SIM (Structured illumination microscopy) has been applied to image desmotubules of ER in primary plasmodesmata (Knox et al., 2015). Research in non-plant systems is demonstrating further potential for super-resolution monitoring of specific MCS proteins. For example, SIM and dSTORM (direct Stochastic Optical Reconstruction Microscopy) were used to visualize clusters of MIRO1 and MIRO2 proteins corresponding to ER-mitochondrial contact sites in mammalian cells (Modi et al., 2019), and ER-PM contact sites were recently investigated using multicolor three-dimensional salvaged fluorescence imaging (Zhang et al., 2020).

### Identifying Novel MCS Components

MCS can be isolated for analysis of lipid and protein content and activity. During cell fractionation and organelle isolation, the presence of MCS leads to the co-purification of membrane regions of interacting organelles, termed “associated membranes.” Andersson et al. (2007) isolated chloroplast-associated fragments of ER membrane from pea protoplasts by fractionation. The lipid composition and polypeptide profiles of these plastid-associated membranes (PLAMS) was distinct from the rest of the chloroplast envelope.

Meta-analyses of organelle proteome datasets could power further discovery of novel MCS components. As MCS can co-purify in organelle isolations as associated membranes, putative candidates might be found among the contaminants identified in organellar proteomes determined by mass spectrometry (Mueller et al., 2017). Moreover, novel proteomics techniques such as the generation of complexomics datasets for specific organelles (Senkler et al., 2017) will allow targeted screening for novel components of specific protein complexes.

Where one contact site component is known, this can be used as the basis for a pull-down assay to identify further proteins within that MCS. Kriechbaumer et al. (2015) performed a GFP-trap assay followed by proteomics using two potential ER-PM contact site proteins (plasmodesmata-localized RTNLB3 and 6) as their bait, thereby identifying 42 and 57 likely interactors for each protein, respectively.

Proximity-labeling has been developed to identify interactors of a protein of interest by biotinylation of its neighbors, separation of these biotin-tagged proteins, and proteomic identification (Figure 1E). APEX2 and BioID are two available biotinylation probes, and both have been used to discover new MCS protein components in mammalian cells (Jing et al., 2015; Lam et al., 2015; van Vliet et al., 2017). BioID has also been used in plants, though not specifically with MCS proteins (Khan et al., 2018). Split variants of both APEX2 (Han et al., 2019) and BioID (De Munter et al., 2017) allow greater specificity through interaction-dependent proximity-labeling, and the former has been applied to ER-PM contact sites in mammalian cells. In plants, established BioID probes show low activity since their optimal working temperature is higher than the plant growth temperature. However, a recently described promiscuous mutant of biotin ligase, TurboID, is less affected by low temperatures compared to earlier BioID variants and has be shown to work efficiently *in planta* (Branon et al., 2018; Arora et al., 2019). Furthermore, a newly reported split-TurboID probe allowed contact-dependent proximity-labelling at ER-mitochondria contact sites in mammalian cells (Cho et al., 2020). Therefore this system offers promise for proximity-labeling in plant MCS as well.

### Detecting and Monitoring MCS With Fluorescent Probes

Studying the interactions of specific protein pairs is possible through FRET (Förster resonance energy transfer) or split fluorescent reporter systems such as BiFC (Bimolecular fluorescence complementation) (Kerppola, 2006; Xing et al., 2016). Both techniques translate proximity into a fluorescent signal, so have potential for visualization of MCS between two organelles. However, fusion partners to detect MCS must be carefully chosen to ensure compatibility with the chosen FP system, specific targeting to the outer membrane of the organelle of interest, and cytosolic orientation of the FP tags.

In BiFC (Figure 1F), a fluorescent signal is emitted if fragments of a fluorescent protein come close enough to allow reassembly and chromophore formation (Magliery et al., 2005). Split fluorescent reporter systems have been successfully applied for MCS detection in animal and fungal cells (Cieri et al., 2018; Shai et al., 2018; Yang et al., 2018) but have been little used in plant systems to date. While they are sensitive tools, spontaneous assembly of BiFC probes can cause false-positive artifacts and non-specific signals. Hence, selection of appropriate negative controls and/or use of ratiometric systems is essential (Grefen and Blatt, 2012; Xing et al., 2016), and protein interactions should be verified by another, independent technique. In most split fluorescent reporter systems, such as split-GFP and YFP-derived split-Venus (Figure 1F), complementation of the fluorophore fragments is irreversible (Magliery et al., 2005), so the duration of the interaction and subsequent dynamics cannot be quantified. This property can, however, be used to deliberately generate “artificial tethers” to manipulate MCS by fixation or expansion. Tao et al. (2019a, b). reported the creation of artificial ER-PM and PM-multivesicular body/tonoplast tethers using split-Venus in plants.

In contrast, FRET (Figure 1G) between a suitable FP pair is fully reversible. Its efficiency depends on the distance between the fluorophores and the specific Förster radius of the FRET pair, covering detection ranges of ∼3–10 nm (for review, see Algar et al., 2019). FRET can be detected by monitoring the acceptor/donor fluorescence intensity ratio, or the decrease in fluorescence lifetime of the donor by Fluorescence Lifetime Imaging (FLIM) (for review, see Xing et al., 2016). Due to its reversibility, FRET can be measured in dynamic systems, though the required imaging time for FLIM (several seconds per image) may limit detection of brief contact events using this approach. One mammalian cell study using rapamycin-inducible tethers, monitored changes in ER-mitochondrial contact site abundance in response to inducer application at high spatial resolution at the organelle level by measuring FRET ratios (Csordás et al., 2010).

FRET-FLIM has been used in plant cells to demonstrate interactions between specific protein partners at MCS in Golgi stacks (Osterrieder et al., 2009). Cells were treated with latrunculin B to depolymerize actin and stop Golgi movement. Some of these interactions occurred only in a subset of Golgi stacks, demonstrating that an *in planta* approach adds additional, spatial information compared to *in vitro*, biochemical studies. In mammalian cells, Venditti et al. (2019) used a FLIM approach to identify ER-Golgi tethers by tagging fluorophores to homogeneously distributed proteins in the membranes of these organelles and systematically depleting tethering candidates by siRNA (small interfering RNA). While the regions of interest on which they conducted their FLIM measurements were not resolved to the level of MCS or even individual Golgi bodies, they paired these measurements with TEM images to examine in detail changes in MCS structures associated with various cell treatments.

Dimerization-dependent FPs (ddFPs; Figure 1H) offer another way to reversibly detect proximity, though they produce low signal levels. While unsuitable for dynamically studying interactions between freely diffusing proteins, ddFPs can be applied to monitor interactions between membrane-associated proteins, as demonstrated with ER-mitochondrial MCS in human and mouse cells (Alford et al., 2012).

FRET, BiFC and ddFPs all depend on the ability of the interaction partners to reach one another, so selecting fusion partners and designing constructs may be problematic if the diameter of the endogenous MCS tethering complex is unknown. Appropriate choice of linker length between the fluorescent tags and proteins of interest is key for successful FRET and split fluorescence assays for protein-protein interactions. Cieri et al. (2018) made two versions of their self-assembling GFP-based probe to detect narrow and wide ER-mitochondrial contact sites, while Várnai et al. (2007) turned this challenge into a tool, using constructs that varied in their linker length to “measure” ER-PM contact distance in mammalian cells.

### Measuring Organelle Tethering Forces

Biophysical methods have been applied to determine physical association between plant organelles. Oikawa et al. (2015) used a femtosecond laser to generate shock waves to quantify the physical interaction between chloroplasts and peroxisomes. This same organelle pairing has been analyzed using optical tweezers (Gao et al., 2016), as have ER-Golgi (Sparkes et al., 2009), ER-chloroplast (Andersson et al., 2007) and ER-mitochondria interactions (White et al., 2020). Optical tweezers provide submicron accuracy to trap and “target” a single organelle and micro-manipulate its position relative to other structures (Figure 1D). In this way, the ER-Golgi tethering function of the Arabidopsis CASP protein was discovered (Osterrieder et al., 2017).

### Demonstrating Molecular Exchange at MCS

To understand the functions of MCS we must identify the processes taking place during organelle contact (Helle et al., 2013). Care must be taken to distinguish MCS-mediated molecular exchange from exchange via alternative mechanisms such as vesicle trafficking (Jouhet et al., 2004, 2019). In the case of protein movement from one organelle to another, fluorescent tags and confocal imaging can demonstrate exchange, though not elucidate its mechanism (Caplan et al., 2015; Thazar-Poulot et al., 2015). Demonstrating exchange of non-protein molecules can present additional challenges as they cannot be directly fluorescently tagged. Genetically encoded ROS biosensors have been used for parallel monitoring of H_2_O_2_ levels in plastids and juxtaposed nuclei in tobacco epidermal cells (Caplan et al., 2015; Exposito-Rodriguez et al., 2017). Jouhet et al. (2004) showed transfer of the glycolipid digalactosyldiacylglycerol (DGDG) from its site of synthesis in plastids to mitochondria in phosphate-deprived plants using biochemical approaches and immunolabelling with EM over time series. Mehrshahi et al. (2013) successfully employed a trans-organellar complementation approach, suggesting biochemical continuity between ER and plastids by mutating enzymes in one organelle, targeting functional versions of each enzyme to the other, and showing that the product of the metabolic pathway was still produced.

## Outlook

Novel microscopy and molecular biology technologies will continue to expand our toolbox for MCS studies. Tracking and localization microscopy (TALM) and Single particle tracking (SPT) techniques, which provide nano-scale information on the localization and movement trajectories of individual FP fusions, have been used to monitor mitochondrial proteins within membrane microdomains in mammalian cells (Appelhans and Busch, 2017) and could similarly be used to monitor functional MCS proteins and/or tethers. Development of reversible split fluorescent probes, such as the recently reported splitFAST (Fluorescence-activating and absorption-shifting tag) system (Tebo and Gautier, 2019), will overcome the challenge of visualizing transient protein interactions in real time. Direct quantification of tethering forces may also become possible through application of genetically encoded, FRET-based force sensors (Freikamp et al., 2016).

Much remains to be learned about the function and composition of MCS, especially in plant systems. Relatively few molecular components of plant MCS have been identified and evidence of the molecular fluxes that they facilitate is similarly sparse (Table 1). Some examples of MCS responses to environmental changes have been recorded, but the molecular details of these responses and their functional significance remain elusive. We anticipate continued rapid progress in this exciting field driven by innovation in microscopic and molecular technologies.

**TABLE 1:** Outcomes of current technologies, summarizing our knowledge to date of organelle interactions and their molecular components in plants.

Organelle pair	Technique	Outcome	References
ER-PM	BiFC	FLS2-VenusN and VenusC-StRem1.3 create artificial ER-PM tethers	Tao et al., 2019a
	Confocal microscopy, FRAP	Ionic stress increases ER-PM contact at SYT1-containing sites	Lee et al., 2019
	Confocal microscopy, FRAP	Plasmolysis of Arabidopsis and N. tabacum cells reduces ER remodeling but does not affect protein flow; ER remains connected to the PM/cell wall via Hechtian strands	Cheng et al., 2017
	Confocal microscopy, TEM	There are ten VAP27 homologs in Arabidopsis; overexpression of VAP27-1 increases ER-PM contact area; VAP27-1 remains at ER-PM-cell wall contacts at the tips of Hechtian strands in plasmolyzed cells	Wang et al., 2016
	Confocal microscopy, FRAP, TEM	SYT1 and VAP27 do not colocalize as reported by Pérez-Sancho et al. (2015) but are associated with distinct, adjacent ER-PM contact sites	Siao et al., 2016
	Confocal microscopy, FRAP, TEM	SYT1 colocalizes with VAP27 at immobile ER-PM contact sites and is important for cellular tolerance of mechanical stress	Pérez-Sancho et al., 2015
	GFP-trap, proteomics, FRET-FLIM	ER-PM contact site components identified by pull-down with existing candidates; FRET-FLIM used to confirm interactors from proteomics	Kriechbaumer et al., 2015
	Confocal microscopy, FRAP	The cytoskeleton, NET3C and VAP27 proteins mediate ER-PM contact in Arabidopsis	Wang et al., 2014
ER-Golgi	Optical tweezers	The Arabidopsis CASP protein mediates ER-Golgi tethering	Osterrieder et al., 2017
	Optical tweezers	Optically trapped Golgi rarely detached from ER, more often causing the ER to remodel, indicating physical attachment	Sparkes et al., 2009
ER-chloroplast	*Trans*-organellar biochemical complementation	Mutating chloroplast-localized proteins and targeting functional versions to the ER still allowed completion of biochemical pathways, suggesting exchange between these organelles, likely via MCS	Mehrshahi et al., 2013
	Optical tweezers, biochemical analysis of isolated MCS fraction	ER associated with a chloroplast was trapped and pulled, but remained attached to the chloroplast at one end in both Arabidopsis and pea cells, indicating physical interaction	Andersson et al., 2007
ER-mitochondrion	Confocal microscopy, optical tweezers	Mitochondria are tethered to the ER in tobacco leaf epidermal cells. Tethering is dependent on Miro2 and affects mitochondrial fusion	White et al., 2020
	Confocal microscopy	ER mediated mitochondrial morphological response to changes in light and cytosolic sugar levels; matrixule formation is ER-dependent	Jaipargas et al., 2015
	Confocal microscopy	Mitochondria associate with the ER in the moss *Physcomitrella patens* and overexpression of the MELL1 protein increases colocalisation	Mueller and Reski, 2015
ER-peroxisome	Fluorescence and confocal microscopy	Live imaging of peroxisomes and the ER in Arabidopsis suggests close association but not luminal continuity	Barton et al., 2013
	Fluorescence and confocal microscopy	Peroxule extension in Arabidopsis is closely aligned with ER tubule dynamics	Sinclair et al., 2009
Peroxisome-oil body	Confocal microscopy, TEM	Sucrose levels within Arabidopsis cells modulate the extent of peroxisome-oil body interactions; the PED3 protein may tether these organelles	Cui et al., 2016
	Confocal microscopy	Peroxisomal extensions facilitate the transfer of the SDP1 protein from peroxisomes to oil bodies in Arabidopsis; the retromer complex may be involved in tethering	Thazar-Poulot et al., 2015
Peroxisome-mitochondria	Confocal microscopy	High light induces peroxule formation, and mitochondria cluster around these structures	Jaipargas et al., 2016
Nucleus-chloroplast	Fluorescence and confocal microscopy	Light-induced chloroplast movement also induces movement of associated nuclei	Higa et al., 2014
	Fluorescence and confocal microscopy, CLEM, Biosensor	Stromule-nuclear association increases during the immune response; protein, and possibly H_2_O_2_, move from the chloroplasts to the nucleus	Caplan et al., 2015
	Fluorescence and confocal microscopy, Biosensor	Demonstration of direct transfer of H_2_O_2_ from plastids to nucleus in tobacco	Exposito-Rodriguez et al., 2017
Chloroplast-peroxisome	Optical tweezers	Peroxisomes are tethered to chloroplasts via peroxules in tobacco leaf epidermal cells	Gao et al., 2016
	Femtosecond laser	In Arabidopsis palisade mesophyll cells, detachment of peroxisomes from chloroplasts requires greater force under light conditions than in the dark	Oikawa et al., 2015
Chloroplast- mitochondrion	Electron microscopy, biochemical analyses	Phosphate-deprived Arabidopsis cells increase chloroplast-mitochondrial contact and transfer digalactosyldiacylglycerol (DGDG) from chloroplasts to mitochondria	Jouhet et al., 2004
PM-tonoplast/multivesicular bodies	BiFC	A wide range of native plant proteins can be used to generate artificial tethering between the PM and tonoplast or multivesicular bodies by fusion to the split-Venus reporter system components	Tao et al., 2019b

## Author Contributions

All authors contributed toward the conception and writing of the manuscript. A-LF generated the figures.

## Conflict of Interest

The authors declare that the research was conducted in the absence of any commercial or financial relationships that could be construed as a potential conflict of interest.
